# Phenotypic characterization of cryptic species in the fungal pathogen *Histoplasma*

**DOI:** 10.1128/msphere.00009-24

**Published:** 2024-05-21

**Authors:** Victoria E. Sepúlveda, Jonathan A. Rader, Jingbaoyi (Janet) Li, William E. Goldman, Daniel R. Matute

**Affiliations:** 1 Department of Biology, University of North Carolina at Chapel Hill, Chapel Hill, North Carolina, USA; 2 Department of Microbiology and Immunology, University of North Carolina at Chapel Hill, Chapel Hill, North Carolina, USA; University of Georgia, Athens, Georgia, USA

**Keywords:** *Histoplasma*, mycology, species

## Abstract

Histoplasmosis is an endemic mycosis that often presents as a respiratory infection in immunocompromised patients. Hundreds of thousands of new infections are reported annually around the world. The etiological agent of the disease, *Histoplasma,* is a dimorphic fungus commonly found in the soil where it grows as mycelia. Humans can become infected by *Histoplasma* through inhalation of its spores (conidia) or mycelial particles. The fungi transition into the yeast phase in the lungs at 37°C. Once in the lungs, yeast cells reside and proliferate inside alveolar macrophages. Genomic work has revealed that *Histoplasma* is composed of at least five cryptic phylogenetic species that differ genetically. Three of those lineages have received new names. Here, we evaluated multiple phenotypic characteristics (colony morphology, secreted proteolytic activity, yeast size, and growth rate) of strains from five of the phylogenetic species of *Histoplasma* to identify phenotypic traits that differentiate between these species: *Histoplasma capsulatum sensu stricto*, *Histoplasma ohiense*, *Histoplasma mississippiense*, *Histoplasma suramericanum*, and an African lineage. We report diagnostic traits for three species. The other two species can be identified by a combination of traits. Our results suggest that (i) there are significant phenotypic differences among the cryptic species of *Histoplasma* and (ii) those differences can be used to positively distinguish those species in a clinical setting and for further study of the evolution of this fungal pathogen.

**IMPORTANCE:**

Identifying species boundaries is a critical component of evolutionary biology. Genome sequencing and the use of molecular markers have advanced our understanding of the evolutionary history of fungal pathogens, including *Histoplasma*, and have allowed for the identification of new species. This is especially important in organisms where morphological characteristics have not been detected. In this study, we revised the taxonomic status of the four named species of the genus *Histoplasma, H. capsulatum sensu stricto* (*ss*), *H. ohiense*, *H. mississippiense*, and *H. suramericanum,* and propose the use of species-specific phenotypic traits to aid their identification when genome sequencing is not available. These results have implications not only for evolutionary study of *Histoplasma* but also for clinicians, as the *Histoplasma* species could determine the outcome of disease and treatment needed.

## INTRODUCTION

*Histoplasma* is an ascomycete dimorphic fungus and the causal agent of histoplasmosis, one of the most common fungal respiratory infections, with hundreds of thousands of new infections occurring annually worldwide (1–4). The disease is particularly common in immunocompromised patients, and the majority of cases are reported in patients who have undergone chemotherapy (5, 6), received an organ transplant (7), or are suffering from acquired immunodeficiency syndrome (AIDS) (8, 9). Histoplasmosis is not mandatorily reported, and for that reason, the true disease burden remains largely unknown (3, 8, 10–12). Nonetheless, there are indications that the disease is more important than is currently understood. Histoplasmin skin reactivity tests suggest that by age 20, more than 90% of individuals residing in endemic regions of the continental United States are skin test-positive for a previous infection or at least exposure to the pathogen (13). Over 100 outbreaks were reported in the 20th century in the USA (14). Similar population assessments indicate that a large proportion of the population has been exposed to *Histoplasma* at some point in their life. Among immunosuppressed patients, the population most at risk, 25% of AIDS patients living in endemic regions of *Histoplasma* develop histoplasmosis; untreated cases usually lead to patient death, and infected individuals often need intense and prolonged antifungal therapy (2, 3).

Initial assessments of diversity in the genus proposed the existence of three different subspecies for *Histoplasma capsulatum. Histoplasma capsulatum* var. *capsulatum* was thought to mainly be found in human patients and caused the classical pulmonary form of histoplasmosis, *H. capsulatum* var. *duboisii* allegedly caused a milder version of the disease with granulomatous lesions in skin and osseous tissues, and *H. capsulatum* var. *farciminosum* was thought to be a pathogen of mules and horses (15). Producing the sexual stage of *Histoplasma* in laboratory conditions is exceedingly difficult (but see references 16–19), which has made the study of potential species boundaries challenging for decades. The application of phylogenetics using molecular markers revealed that the three initially proposed lineages were artifactual and did not follow the evolutionary history of the pathogen (20, 21). Multilocus sequence typing revealed at least eight genetic clusters within *Histoplasma*: North American 1 clade (NAm 1), North American 2 clade (NAm 2), Latin American A clade (LAm A), Latin American B clade (LAm B), Australian clade, Netherlands clade, Eurasian clade, and African clade (21). Another classification of *Histoplasma* is based on the presence/absence of the polysaccharide α-(1, 3)-glucan in the cell wall (*AGS1* locus), produced only during the yeast phase. Strains that possess α-(1, 3)-glucan have a rough colony morphology and are classified as chemotype 2 strains, which represent most of the strains found worldwide. Strains that lack α-(1, 3)-glucan have a smooth colony morphology, are classified as chemotype 1 strains, and are restricted to a North American lineage (22–26). The virulence requirements for α-(1, 3)-glucan have been shown to differ among *Histoplasma* lineages (26). Additional, as-yet unidentified lineages are likely to exist within *Histoplasma* (21, 27).

The implementation of genome sequencing confirmed the existence of differentiated genetic lineages and revealed that these clades were sufficiently diverged to be considered phylogenetic species (28–30). Five species satisfied the first assessment of genome concordance and differentiation: *Histoplasma ohiense*, *Histoplasma mississippiense*, *Histoplasma capsulatum sensu stricto (ss*), *Histoplasma suramericanum*, and a *Histoplasma* lineage from continental Africa. Additional genome sequencing revealed the existence of two additional phylogenetic species, one endemic to the Indian subcontinent (30) and one endemic to southern Brazil (29). These seven species in the *Histoplasma* genome diverged over 1.5 million years ago and have accrued extensive genetic differences that make them advanced along the speciation continuum (28).

The taxonomic rearrangement of the *Histoplasma* genus set the basis for further studies and propelled important developments in understanding the biology of *Histoplasma*. Genome assembly of strains from each of these species suggested genome content differences and rearrangements which, in turn, have suggested a rapid turnover of genome structure in the genus (31). Surveys of gene exchange have also revealed low levels of admixture among lineages, which indicates that hybridization might be of importance in the evolution of *Histoplasma* (30, 32). From a more applied perspective, Sepúlveda et al. (28) reported extensive genetic differences along the genome and the possibility of using molecular markers for molecular detection, which could be harnessed by clinical researchers and inform the epidemiological patterns of each of these lineages.

Despite all the genomic progress, no systematic assessment has been performed to determine whether these phylogenetic species differ phenotypically. Clearly, there is extensive genetic differentiation in the genus, even in the face of extensive geographic overlap (33), but taxonomic revisions should be accompanied by descriptions that can serve clinical and evolutionary researchers alike (34, 35). The initial species description suggested that previous assessments of phenotypic differentiation in *Histoplasma* might follow species boundaries (28). Nonetheless, no survey has measured potential intraspecific and interspecific phenotypic variation in common conditions. Here, we bridge that gap. We explored whether the genetic differentiation within *Histoplasma* might explain some variability in the group and whether phenotypic variation follows species boundaries. In this report, we quantified four phenotypic traits and found yeast culture-based diagnostic characters for three of the *Histoplasma* species, *H. ohiense*, *H. mississippiense*, and *H. suramericanum*. The other two species, *H. capsulatum* and the African lineage, can be identified by a combination of multiple traits. We also developed a PCR and restriction enzyme-based assay that allows for discrimination among all these *Histoplasma* species. Using this information, we revise the taxonomic status of the named species of the genus *Histoplasma*.

## MATERIALS AND METHODS

### Fungal strains and culture conditions

*Histoplasma* isolates used in this study were donated to William E. Goldman during a span of 15 years. Information pertinent to each isolate is listed in Table S1. All isolates were kept in 15% glycerol at −80°C until they were ready to be subcultured. An aliquot of the frozen culture was streaked into *Histoplasma* macrophage medium (HMM) plates. Strains were then grown in HMM (solid or liquid) at 37°C with 5% CO_2_ as previously described (36). Solid medium contained 0.6% agarose (SeaKem ME grade) and 25 mM FeSO4. All liquid cultures were incubated at 37°C with 5% CO_2_ on an orbital shaker (Infors HT Multitron) at 150 rpm. All reference strains were deposited in the Westerdijk Fungal Biodiversity Institute CBS collection (Table S1).

### Yeast colony morphology

We scored the yeast colony morphology of 27 *Histoplasma* strains (at least three isolates from each species, Table S1). For each isolate, we added 10 µL of a late exponential phase culture on a HMM plate. We grew 36 aliquots per Petri dish and incubated the plates at 37°C in 5% CO_2_ for at least 10 days before we imaged each colony. Colonies were classified as rough or smooth. To ensure reproducibility, we scored at least 12 colonies per species, but no isolate showed variation in colony morphology.

### Evaluation of extracellular proteolytic activity

The second trait we evaluated was proteolytic activity. Several studies have reported the existence of extracellularly secreted serine proteases in *Histoplasma*. In particular, isolates from the RFLP1 group (later named *H. mississippiense*) were the only ones that manifested this phenotype (37; cf. reference 38 for reports of proteolytic activity in African strains). To evaluate extracellular proteolytic activity in different species of *Histoplasma*, we grew 27 *Histoplasma* strains (Table S1) in HMM plates supplemented with 1.5% skim milk. Strains with proteolytic activity show a clear halo around their yeast colonies. Fifteen grams of instant nonfat dry milk (Hoosier Hill Farm brand, Middleton, WI) were reconstituted in 500 mL of distilled water. Once the skim milk was fully dissolved, 6 g of agarose (SeaKem ME grade) was added and autoclaved to make HMM plates as previously described (36). Ten microliters of a late exponential phase culture were spotted on HMM plates supplemented with skim milk. We spotted four strains per plate to allow for any transparent clearance area around fungal spots to appear, indicative of proteolytic activity. We incubated the experiment using the same conditions as described immediately above to study yeast colony morphology. We scored at least six colonies per isolate for the presence/absence of a halo (range between 6 and 12 colonies), and when present, we measured halo size. The size of the halo was measured as the distance from the edge of the colony to the outer edge of the cleared ring. To compare halo sizes, we used a one-factor linear model (function anova, library stats [39]).

### Optical density and growth curves of *Histoplasma* yeast cultures

We also measured the growth rate of different *Histoplasma* genotypes in liquid media for 12 strains (growth curves were performed at least twice per strain). Table S1 lists the strains used for this experiment. For the growth curves, we inoculated 30 mL of HMM broth with 1 × 10^6^ yeast/mL and grew the culture for 11 days. We removed 600 µL from each culture and mixed them with 300 µL of 3M NaOH in a plastic cuvette, which was covered with Parafilm and vortexed for 10 seconds to separate yeast clumps and measure optical density (OD) in a GENESYS 10vis spectrophotometer (Thermo Spectronic) starting at day 0, and at every 24 hours after that until day 11. To quantify the rate of growth, we used a four-parameter logistic model with the following form:


(1) $$OD∼d+(a-d)/[1+(time/c){]}^{b}$$

where *a* is the OD at the beginning of the experiment (presumably close to zero), *b* is the rate of increase in OD at point *c*, the inflection point of the curve, and *d* is the maximum OD in the curve, the asymptote. This model allows for an initial growth where cells are dividing but do not increase the OD value and includes an asymptote, calculated from the data, at which cells do not replicate anymore. Since nonlinear logistic regression has difficulties optimizing the values for each of the four constants in the equations, we tried 10 starting values per constant and found the model with the lowest Akaike information criterion (AIC) (40) with the function AIC (library stats [39]). To fit the regressions, we pooled isolates within phylogenetic species.

To determine whether the four fitted parameters differed among species, we generated 1,000 bootstrapped regressions using the R function nls.boot (library nlstools [41, 42]). We then compared the values of *b*, *c,* and *d* across species using non-parametric tests (Wilcoxon rank sum test with continuity correction, function wilcox.test, library stats [39]).

### Yeast area

Finally, we studied the area of individual yeast cells in different *Histoplasma* isolates. We scored at least two isolates per *Histoplasma* species to evaluate phenotypic variability between species. In total, we scored 12 strains (listed in Table S1). We mixed 10 µL from a yeast culture that had large yeast clumps removed with 10 µL of Lactophenol Cotton Blue on a glass slide. Differential interference contrast (DIC) images were obtained using 100X/1.4 Oil UPlan S Apo PSF quality objective on an Olympus BX-61 microscope and collected using a QImaging RETIGA 4000R color camera and Volocity 6.3 acquisition software. Exposure was adjusted to ensure pixel intensities were not saturated (pixel size: 0.0608 µm/pixel). We then measured yeast cell area by drawing an ellipse around each imaged cell in imageJ (43). We used the ellipse area (in μm^2^) as a measure of cell size. To compare the yeast cell size across different species, we used a linear model (LM) in which cell area was the response and the species identity was the grouping factor. We used the R function aov (library stats [39]). Finally, we compared among lineages (all pairwise comparisons) using Tukey contrasts with the R function TukeyHSD (library stats [39]).

### Principal component analyses

To visualize the morphological differentiation between lineages, we used a principal component analysis (PCA). We included the 12 isolates for which we had measured the four traits described above. To calculate the correlation between variables, we used the function cor (library stats [39]). To visualize the matrix in a heatmap, we used the function ggcorrplot (library ggcorrplot [44]). We then used the function PCA (library FactoMineR [45, 46]). We extracted the contributions of each variable to the PCs using the function get_pca_var. To generate a biplot of the first two PCs and the contributions of each trait, we used the function fviz_pca_biplot (library Factoextra [47]). The first two PCs explained the majority of the variance in the data set (see Results), and for that reason, we did not explore further PCs.

### Identification of *Histoplasma* species by PCR and restriction enzymes

We used polymerase chain reaction (PCR) in combination with restriction enzymes to develop a diagnostic assay that discriminates among the *Histoplasma* species analyzed in this study based on previous surveys of genetic polymorphism in *Histoplasma* (20, 21). We designed primers (forward primer 5′- TTT AAA CGA AGC CCC CAC GG-3′ and reverse primer 5′- TGC ATC AGC CGT AGT AAT AGG TTC CG) to amplify a 1.5 kb region of the delta-9 fatty acid desaturase gene. We used the Wizard SV Gel and PCR clean-up system (Promega) to purify each individual PCR product. We used single restriction enzymes (New England Biolabs) using five different enzymes (BamHI, XhoI, StuI, BsrGI, and BanII) to digest the 1.5 Kb PCR fragment and generate a restriction pattern that would allow us to discriminate among the different *Histoplasma* species. We visualized the restriction products in 1% agarose gels. To visualize the BanII digestion products, we used 0.8% and 2% agarose gels.

## RESULTS

### *Histoplasma ohiense* differs in their yeast colony morphology

**Fig 1:**
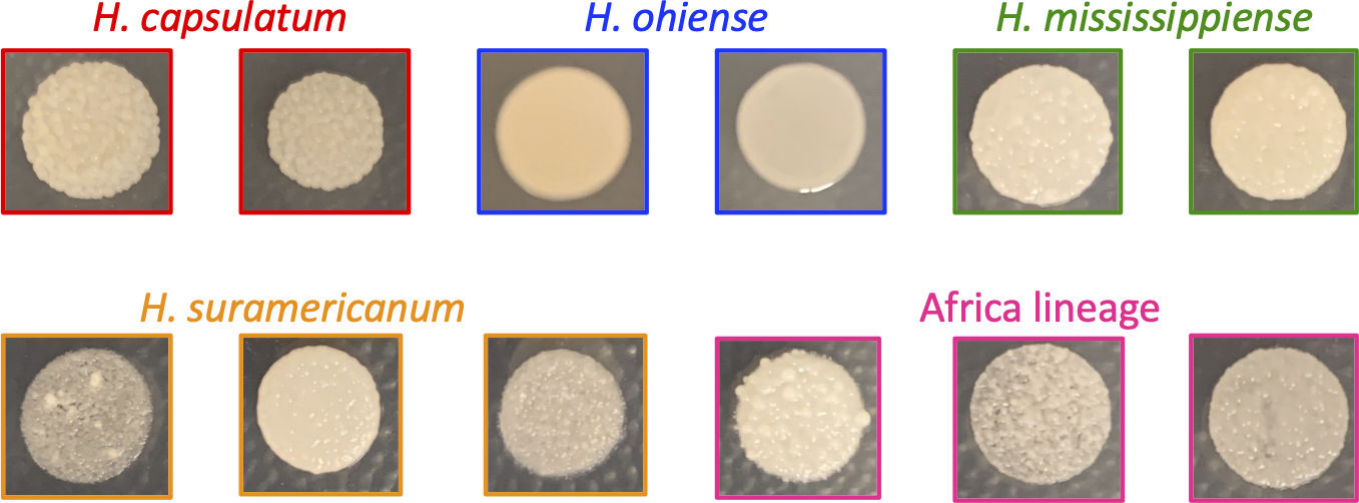
Colony morphology in *Histoplasma* species. Fungal strains were grown on agarose-solidified HMM plates. Smooth morphology occurs in the absence of α-(1, 3)-glucan in the cell walls. *H. ohiense* is the only species that shows a smooth colony morphology due to the lack of α-(1, 3)-glucan.

Multiple previous studies have reported variation in yeast colony morphology across isolates of *Histoplasma* (22–24). Some isolates show smooth colonies, whereas others show rough ones. We studied whether this phenotypic variation was species-specific or whether there was intraspecific variation within five phylogenetic species of *Histoplasma*. Figure 1 shows the yeast colony morphology for 12 representative *Histoplasma* isolates after growing at 37°C for 10 days in HMM. All isolates from three *Histoplasma* species, *H. capsulatum* (*N* = 2), *Histoplasma suramericanum* (*N* = 3), and the Africa clade (*N* = 3), had rough yeast colonies (at least six colonies per isolate). Eight of the nine isolates of *H. mississippiense* (six colonies per isolate) had rough colonies. The only exception was the Downs strain, a strain isolated in 1968 and shown to be avirulent in mice (48, 49), which showed smooth colonies. All the replicates across isolates of *H. ohiense* (*N* = 10 isolates) had smooth yeast colonies. This morphological difference within *Histoplasma* has been attributed to the lack of α-(1, 3) glucan in their cell wall (25, 50–52). These comparisons indicate that yeast colony morphology is a diagnostic trait of *H. ohiense* and is sufficient to differentiate the species from the other four lineages.

### Production of extracellular proteolytic activity is restricted to *H. mississippiense*

**Fig 2:**
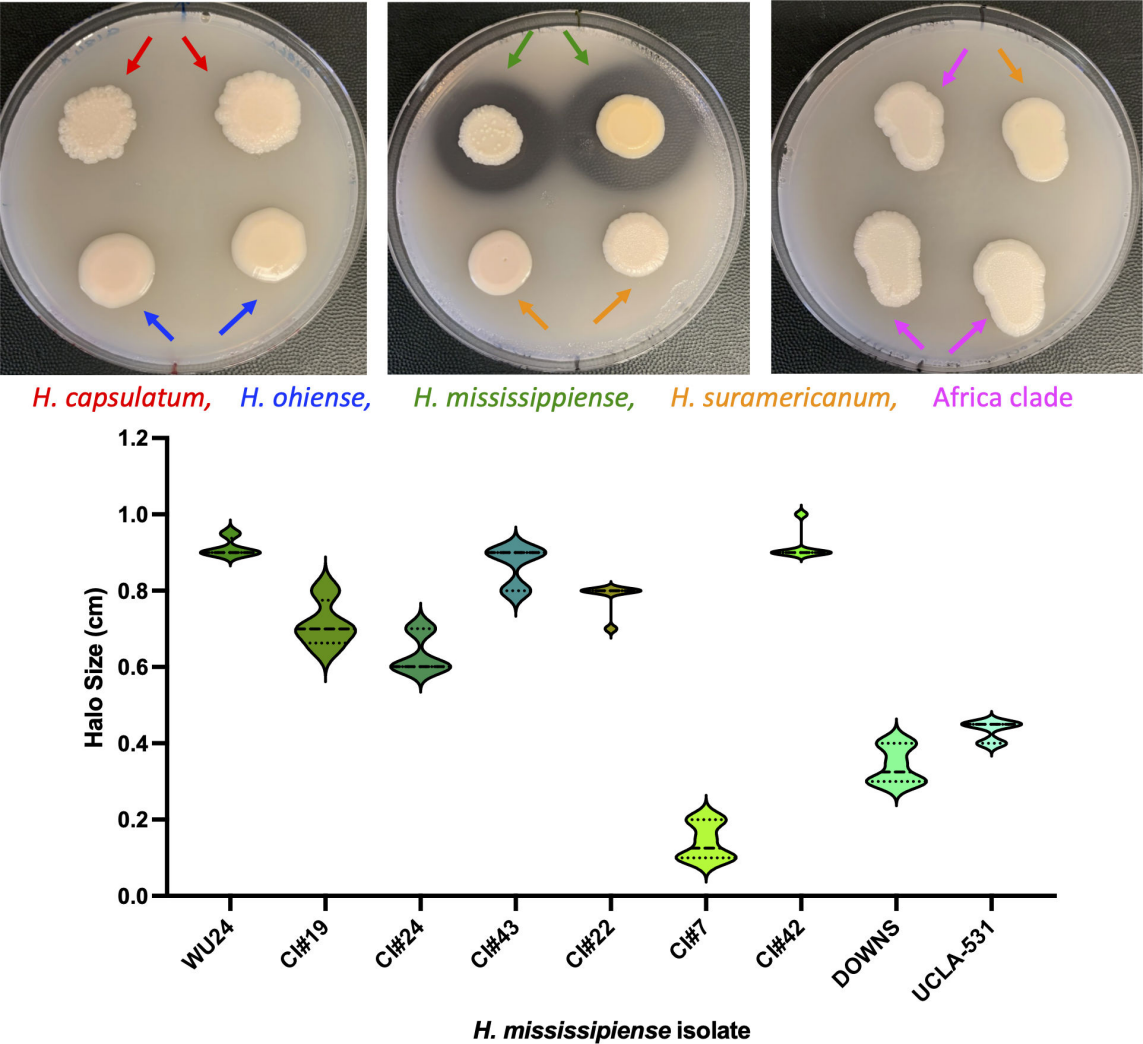
Demonstration of extracellular proteolytic activity in *Histoplasma* isolates. Fungal strains were grown on agarose-solidified HMM supplemented with 1.5% skim milk. Secreted proteolytic activity is visible as transparent clearance halos around fungal colonies and was assessed after 10 days of growth at 37°C in 5% CO_2_. The presence of extracellular proteases was observed only in *H. mississippiense* strains (top, middle panel). The size of the halo varied within *H. mississippiense* (bottom panel). The other four species of *Histoplasma* included in this report showed no proteolytic activity (i.e., no halo; right and left top panels, and data not shown). Bottom panel: dotted lines represent the 75^th^ (top) and 25^th^ (bottom) percentiles. The dashed line is the median.

Production of extracellular proteolytic activity using HHM media supplemented with skim milk had been previously described in some isolates of *Histoplasma* (37). We studied whether the five different species differed in their proteolytic ability. We grew five of the previously identified lineages in skim milk media to determine whether they showed proteolytic activity. Figure 2 shows an example of each of the five species growing as yeast in HMM media supplemented with skim milk at 37°C. Of the five species, *H. mississippiense* was the only lineage to show a clearance halo, which is a proxy of the ability of the colony to break down proteins. Within *H. mississippiense*, the mean halo size ranged between 0.142 cm (CI7) and 0.917 cm (CI42; Fig. 2B), a difference that was significant (LM:F_8,61_ = 17.122 P = 5.028 × 10^−13^). The observation is consistent with previous studies which suggested that isolates from this lineage (originally termed RFLP1) are the only ones with an extracellular protease ability (37) and that proteolytic activity is a diagnostic trait of *H. mississippiense*.

### Growth curves and optical density

**Fig 3:**
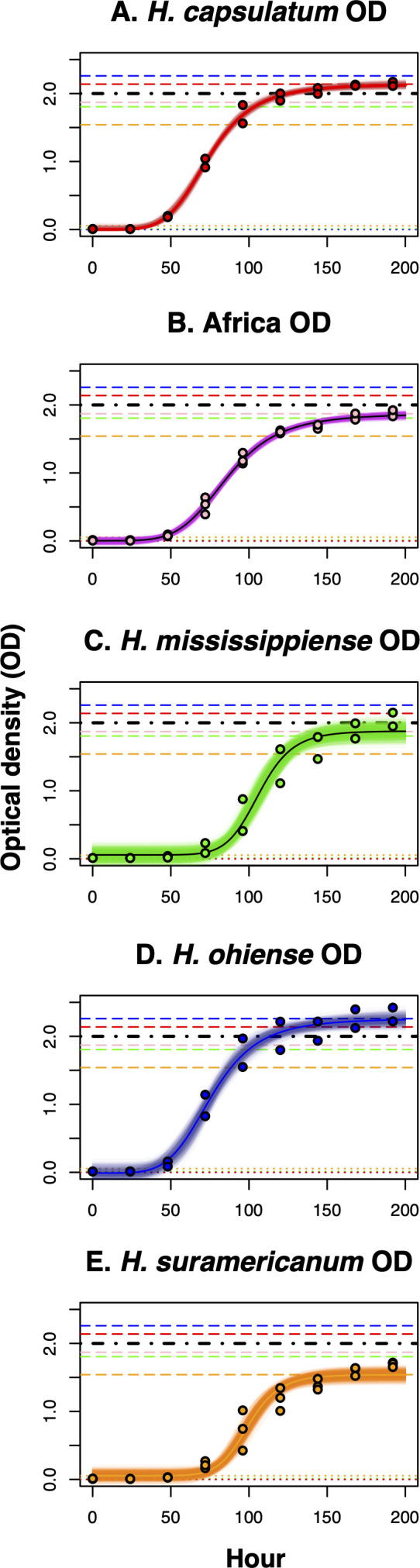
Four-parameter logistic models for the rate of growth of five *Histoplasma* species. All experiments were done in HMM broth media at 37°C with 5% CO_2_. Growth was measured by recording the optical density (OD600) of liquid cultures at different time points (0, 24, 48, 72, 120, 144, 168, and 192). (A) *H. capsulatum*. (B) African lineage (C) *H. mississippiense* (D) *H. ohiense* (E) *H. suramericanum*. Semitransparent lines show 1,000 bootstrapped model fits.

We evaluated whether different genotypes of *Histoplasma* had differences in their growth rate and if such differences corresponded with species boundaries. We used optical density as a proxy for the number of cells in a liquid culture and fitted logistic models that modeled the rate of increase of the different species. Figure 3 shows the results of the best fit for each species. The growth curves of the five species show a better fit to a logistic dose-response function than to a linear function (Table S2). Non-linear regressions are highly dependent on the seed values used for the optimizations; hence, we maximized the fit using AIC values (Table S3).

**Fig 4:**
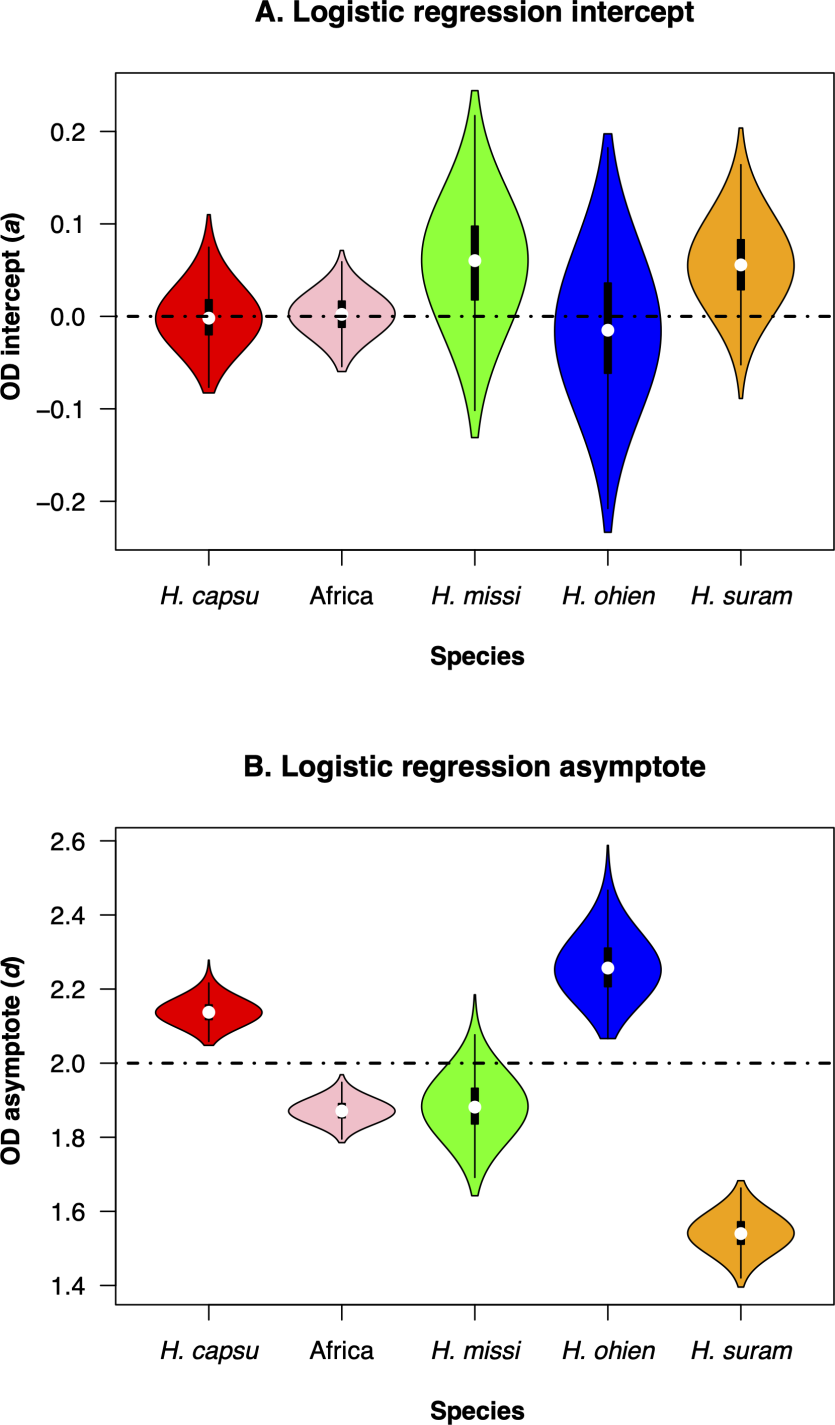
Intercept and asymptote distributions of bootstrapped regressions. (A) Intercept (*a* in equation 1). (B) Asymptote (*d* in equation 1). Each boxplot shows 1,000 values of bootstrapped non-linear regressions (shown as semitransparent lines in Fig. 3). *capsu: H. capsulatum* ss, *H. missi: H. mississippiense*, *H. ohien: H. ohiense*, *H. suram: H. suramericanum*.

We focused on two of the four calculated parameters, the intercept (*a*) and the asymptote (*d*). We found that although there are significant differences among bootstrapped distributions of the intercept (*a* Table 1), all intercepts are also centered around zero (Fig. 4A). Since the intercepts were similar, comparisons among asymptote (d) values are informative and indicate whether the species have differences in the growth saturation point. Indeed, the values of all the inferred asymptotes differed among the five species, but there were two clearly differentiated groups (Table 1). The growth curves at 264 hours for two of the species (*H. capsulatum ss* and *H. ohiense*) had OD asymptotes higher than 2. On the other hand, the other three species (*H. mississippiense,* the African lineage, and *H. suramericanum*) had OD asymptotes lower than 2 (Fig. 4B; Table 1). This difference can be used to discriminate between these two clusters of species and suggest that OD-based growth curves can be a taxonomic trait that can aid species identification in *Histoplasma*, but one that does not serve as a diagnostic trait in isolation.

**TABLE 1:** Pairwise comparisons between two of the parameters of the logistic regression, *a* and *d*^*a*^

Parameter	Species	Estimate	SE (bootstrap)	Species				
				*capsu*	Africa	*missi*	*ohien*	*suram*
*a*	*capsu*	9.224 × 10^−5^	9.312 × 10^−4^	*	5.338 × 10^−4^	5.68 × 10^−10^	<1 × 10^−10^	<1 × 10^−10^
	Africa	2.906 × 10^−3^	6.539 × 10^−4^	4,54,346	*	4.775 × 10^−3^	<1 × 10^−10^	<1 × 10^−10^
	*missi*	5.708 × 10^−2^	1.903 × 10^−3^	4,19,071	4,62,616	*	3.57 × 10^−7^	<1 × 10^−10^
	*ohien*	−1.128 × 10^−2^	2.272 × 10^−3^	2,96,769	3,70,934	4,33,831	*	<1 × 10^−10^
	*suram*	5.461 × 10^−2^	1.294 × 10^−4^	1,30,447	2,16,910	2,92,041	2,96,485	*
*d*	*capsu*	2.138	9.654 × 10^−4^	*	<1 × 10^−10^	<1 × 10^−10^	<1 × 10^−10^	<1 × 10^−10^
	Africa	1.871	8.610 × 10^−4^	9,98,001	*	<1 × 10^−10^	<1 × 10^−10^	<1 × 10^−10^
	*missi*	1.879	2.318 × 10^−3^	9,98,001	9,97,996	*	<1 × 10^−10^	<1 × 10^−10^
	*ohien*	2.261	2.390 × 10^−3^	9,93,006	2,96,455	2	*	<1 × 10^−10^
	*suram*	1.541	1.427 × 10^−3^	9,98,001	9,98,001	9,96,801	9,98,001	*

^*a*^ *a* corresponds to the intercept; *d* corresponds to the asymptote. The lower triangular matrix shows the *W* from the Wilcoxon test. Upper triangular matrix shows the *P*-value. Each parameter estimate is estimated from the non-linear regression; the standard error (SE) was calculated from the distribution of the 1,000 bootstrap samplings shown in Fig. 3. *capsu*: *H. capsulatum* ss; Africa: African lineage; *missi*: *H. mississippiense*; *ohien*: *H. ohiense*; *suram*: *H. suramericanum*. * marks the diagonal.

### Yeast cell size differs among *Histoplasma* species

We also scored the area of yeast cells across lineages of *Histoplasma*. We found cell size variation among species (LM, species effect: *F*_12,1688_ = 13.487, *P* < 1 × 10^−10^). Table 2 shows all the pairwise comparisons among species. *H. ohiense* had a larger cell size than the other four species (Table 2). On the other hand, *H. suramericanum* had a smaller cell size than all the other species (Table 2). Notably, we also found heterogeneity across isolates (LM, isolate effect nested within species: *F*_12,1688_ = 3.378, *P* = 6.723 × 10^−5^). These results indicate that although there is phenotypic variation within species, yeast cell size might serve as a diagnostic trait for *H. ohiense*, but not for the remaining *Histoplasma* species.

**TABLE 2:** Tukey HSD pairwise comparisons show that *H. ohiense* has a smaller yeast cell size than other species of *Histoplasma^a^*

Species	Mean (μm^2^)	SD (μm^2^)	Tukey’s HSD tests				
			*capsu*	Africa	*missi*	*ohien*	*suram*
*capsu*	3.907	2.666	*	0.988	0.994	0.008	< 0.001
*Africa*	4.112	3.199	0.098	*	0.999	0.015	< 0.001
*missi*	3.995	2.314	0.087	0.011	*	0.022	< 0.001
*ohien*	4.965	6.259	0.669	0.571	0.581	*	< 0.001
*suram*	3.142	1.856	0.766	0.864	0.365	1.434	*

^*a*^ SD: standard deviation. *capsu*: *H. capsulatum* ss; Africa: African lineage; *missi*: *H. mississippiense*; *ohien*: *H. ohiense*; *suram*: *H. suramericanum*. * marks the diagonal.

### Principal component analysis

**Fig 5:**
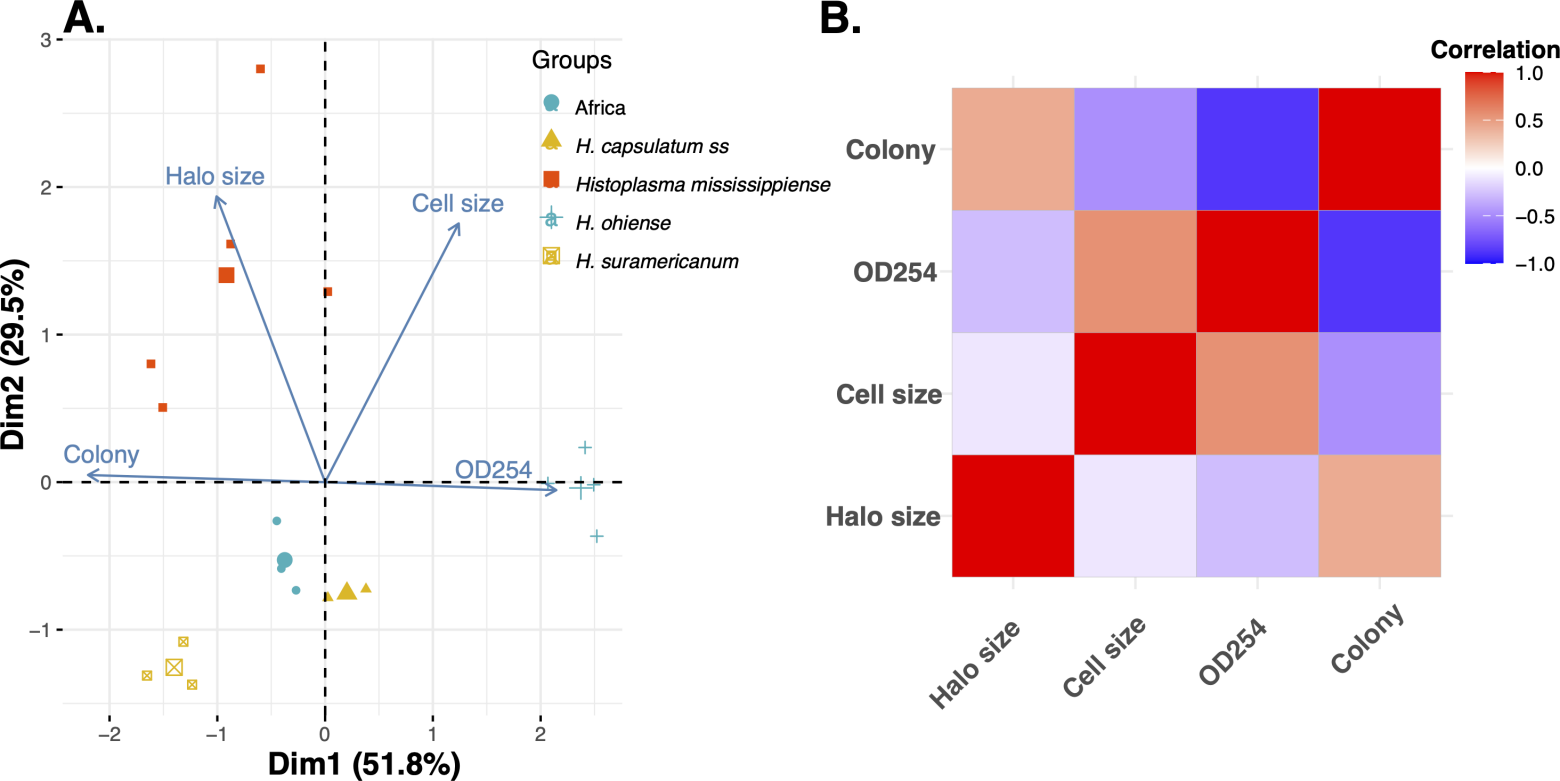
A synthesis of the phenotypic differentiation among *Histoplasma* species. (A) Principal component analysis suggests that the five species of *Histoplasma* in this study differ in their phenotypes. The larger points represent the mean location for each of the five phylogenetic species. (B) Correlation coefficients between the four studied traits. Table S4 shows the contributions of each trait to the two PCs.

Next, we synthesized the array of morphological traits using a principal component analysis to determine what combination of traits distinguishes these lineages most effectively (Fig. 5A). Table S4 shows the contributions of each trait to each of the PCs. Figure S1 shows a scree plot with the contributions of the four PCs. PC1, which encompasses 51% of the phenotypic variance, is mostly explained by colony morphology and optical density after 264 hours. The contributions of these two traits to PC1 are similar and are on the order of ~40%. The two traits are diagnostic of *H. ohiense* and are strongly correlated (Fig. 5B). PC2 is largely explained by the presence of a proteolytic halo and cell size. The former is a diagnostic trait of *H. mississipiense*, the latter differentiates *H. ohiense* and *H. suramericanum* from the other three species. Two species, Africa and *H. capsulatum ss,* are in close proximity on the PC axes, which reflects their phenotypic similarity. Nonetheless, optical density in liquid culture can effectively discriminate between these two lineages. Overall, the first two PCs collectively explain 84% of the phenotypic variance and show that the combination of these four traits effectively differentiates among the five species.

### PCR and restriction enzyme-based assay allows to discriminate among *Histoplasma* spp

**Fig 6:**
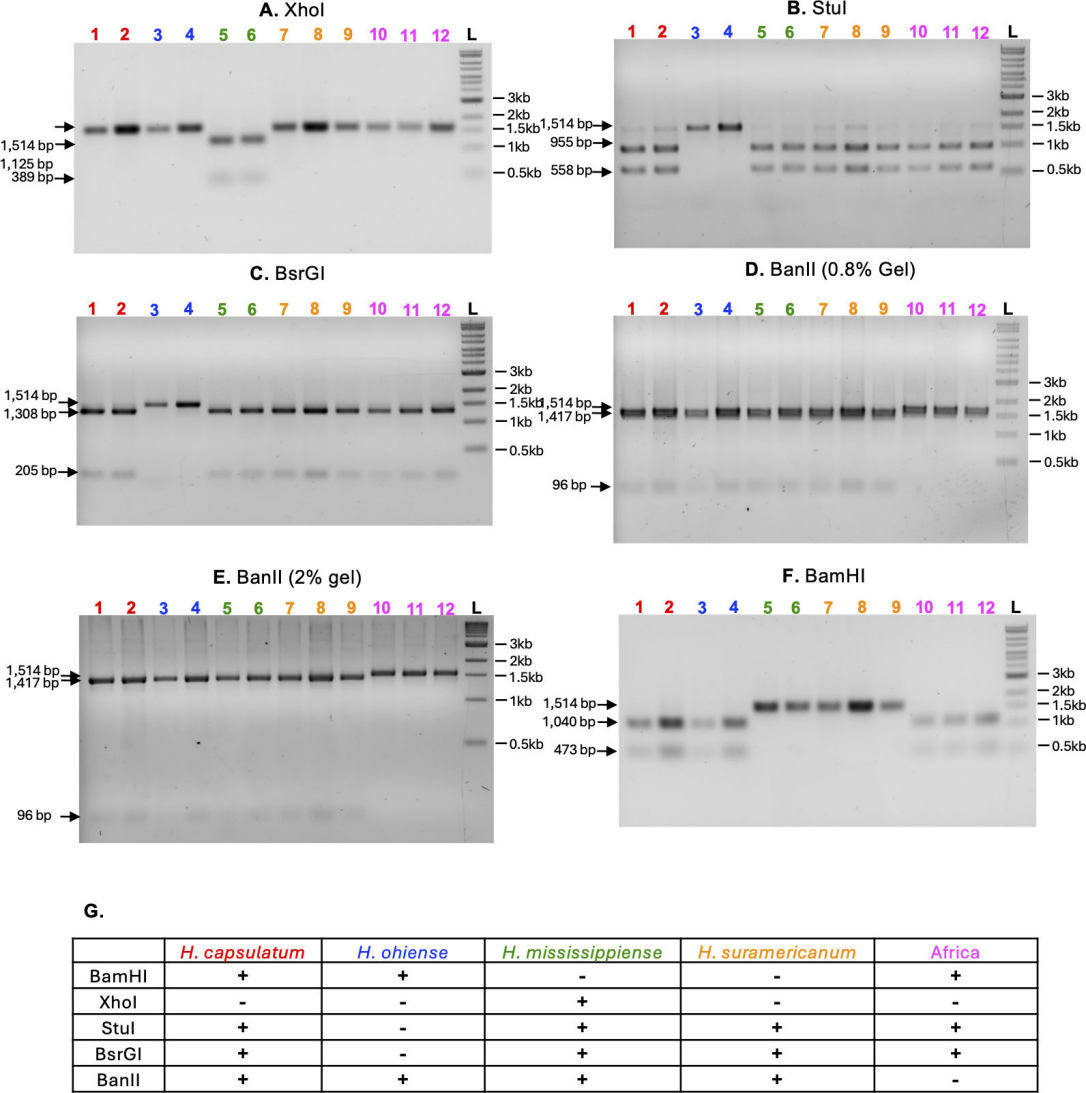
PCR and restriction enzyme diagnostic assay to discriminate among *Histoplasma* species. A total of 1.5 kb PCR fragments of the delta-9 fatty acid desaturase gene from all reference strains were digested with XhoI (A-), StuI (B), BsrGI (C), BanII (D and E), and BamHI (F). Red: *H. capsulatum ss* (1. G186A, 2. G184A); blue: *H. ohiense* (3. G217B, 4. CI#17); green: *H. mississippiense* (5. WU24, 5. CI#19); orange: *H. suramericanum* (7. 3/11, 8. 21/14, 9. 27/14); and magenta: Africa (10. H88, 11. H143, 12. *H. duboisii*); DNA ladder (L. Quick-Load 1 kb Extend DNA Ladder, NEB). (G). A summary of the digestion patterns of all species indicating the presence (+) or absence (−) of a restriction site for the respective restriction enzyme used.

Finally, we developed a diagnostic assay to discriminate between the five *Histoplasma* species included in this report. We used DNA from 27 strains to amplify a 1.5 kb region of the delta-9 fatty acid desaturase gene. Figure 6 shows the PCR restriction patterns from a set of representative strains. Figure S2 shows additional strains of *H. mississipiense* and *H. ohiense*. Three species can be identified with a single DNA digestion. *Histoplasma mississipiense* strains are the only ones with an XhoI restriction site within the amplified region, resulting in a 1,125 bp and a 389 bp bands (Fig. 6A). *H. ohiense* are the only strains that show no digestion with either StuI or BsrGI. Digestions with StuI produce 955 bp and 558 bp bands in all other species (Fig. 6B), whereas digestions with BsrGI produce 1,308 bp and 205 bp bands in all other species (Fig. 6C). Strains from the African clade show no digestion with BanII, whereas digestions with the same enzyme produce two fragments (1,417 bp and 96 bp) in all the other four *Histoplasma* species (Fig. 6D and E). *Histoplasma suramericanum* can be identified with two restriction enzymes as they are the only isolates that show no digestion with BamHI or XhoI. The two enzymes produce two bands in some but not all other species (BamHI: 1,040 bp and 473 bp, Fig. 6F; XhoI 1,125 bp and 389 bp, Fig. 6A). Finally, *H. capsulatum ss* can be identified by a combination of restriction sites: the presence of a BamHI, StuI, BsrGI, and BanII and the absence of a XhoI restriction site. Figure 6F summarizes the restriction patterns for each species.

### Taxonomy

Table 3 and Figure 5 summarize the results of our phenotypic surveys. The combination of these traits is sufficient to differentiate between the five cryptic species of *Histoplasma*. Using these phenotyping surveys, we re-describe the three named species of *Histoplasma.*

**TABLE 3:** Phenotypic differences among five different species of *Histoplasma^a^*

	Trait			
Species	Yeast colony morphology	Proteolytic activity	Yeast cell size	OD after 264 hours
*H. ohiense*	Smooth	No	Large	>2.0
*H. mississippiense*	Rough	Yes	Medium	<2.0
*H. suramericanum*	Rough	No	Small	<2.0
*H. capsulatum ss*	Rough	No	Medium	>2.0
Africa	Rough	No	Medium	<2.0

^*a*^ Species diagnostic traits are underlined.

*Histoplasma mississippiense* V.E. Sepúlveda, R. Márquez, Turissini, W.E. Goldman & Matute, sp. nov. MB 853752.

For a detailed description, see Sepúlveda et al., mBio 8(6): e01339-17, 13 (2017).

Holotype: CBS 145498 preserved in a metabolically inactive state.

Previously published as *Histoplasma mississippiense* V.E. Sepúlveda, R. Márquez, Turissini, W.E. Goldman & Matute, mBio 8(6): e01339-17, 12 (2017), nom. inval., Art. 40.7 (Shenzhen), MB 823360]

*Histoplasma ohiense* V.E. Sepúlveda, R. Márquez, Turissini, W.E. Goldman & Matute, sp. nov. MB 853781.

For a detailed description, see Sepúlveda et al., mBio 8(6): e01339-17, 13 (2017).

Holotype: CBS 145496 preserved in a metabolically inactive state.

Previously published as *Histoplasma ohiense* V.E. Sepúlveda, R. Márquez, Turissini, W.E. Goldman & Matute, mBio 8(6): e01339-17, 12 (2017), nom. inval., Art. 40.7 (Shenzhen), MB 823361]

*Histoplasma suramericanum* V.E. Sepúlveda, R. Márquez, Turissini, W.E. Goldman & Matute, sp. nov. MB 853753.

For a detailed description see Sepúlveda et al., mBio 8(6): e01339-17, 13 (2017).

Holotype: CBS 145499 preserved in a metabolically inactive state.

Previously published as *Histoplasma suramericanum* V.E. Sepúlveda, R. Márquez, Turissini, W.E. Goldman & Matute, mBio 8(6): e01339-17, 12 (2017), nom. inval., Art. 40.7 (Shenzhen), MB 823362]

## DISCUSSION

Identifying species boundaries has been a challenge in microbial eukaryotes because producing sexual stages and making direct measurements of reproductive isolation, the signature of speciation, are usually impractical and often unfeasible (reviewed in references 35, 53–55). Measuring the extent of genetic divergence, and identifying reductions in gene flow, has been a powerful substitute to uncover cryptic speciation in fungal pathogens (56, 57). The incorporation of genomics has opened the door to describing the evolutionary processes that govern speciation and trait diversification in fungal pathogens (34, 53). Nonetheless, genome sequencing alone might be impractical for the identification of pathogens, particularly in clinical settings. In this study, we report phenotypic differences that are sufficient to identify five phylogenetic species of *Histoplasma* and revise their taxonomic status. In particular, we report that *H. ohiense* can be identified by its characteristic smooth colonies and larger cell size, and *H. mississippiense* by its extracellular proteolytic activity. *H. suramericanum* shows a smaller cell size than all the other species. The other two species, *H. mississippiense* and the African lineage, differ in their OD600 at 264 hours. Additionally, we developed a PCR and restriction enzyme-based assay that allows for the differentiation of the five included phylogenetic species. The five species can also be discriminated with other PCR probes (20, 21, 28). Our results are of importance to evolutionary and clinical mycologists alike because the diagnosis of species boundaries is the first step to understanding evolutionary dynamics, broadly defined, and could shed light on the evolution of different virulence mechanisms, antifungal resistance, and clinical traits.

Other studies have reported differences in the morphology of *Histoplasma* isolates and among clusters of genotypes. Okeke and Muller (58) described the presence of extracellular collagenolytic proteinases by *H. capsulatum* var. *duboisii* and *H. capsulatum* var. *capsulatum*. Since these classifications do not follow a phylogenetic framework (20, 21), the results are not immediately comparable. Importantly, our results are consistent with Zarnowski et al. (37), where the extracellularly secreted serine protease activity was restricted to *H. mississippiense* isolates (formerly known as RFLP class one or NAm 1 clade, 20, 21, 59). The role of the extracellularly secreted serine protease activity in *H. mississippiense* virulence remains unexplored. Muotoe-Okafor et al. (38) detected a similar proteolytic activity in a cluster of African samples. To date, *H. mississippiense* has not been collected in Africa, and these results might indicate that some African isolates, but not the ones included in this study, might have serine protease activities similar to the ones in *H. mississippiense*. We offer three potential explanations for these results. First, the African samples with serine protease might belong to a lineage closely related to *H. mississippiense* or *H. mississippiense* themselves. Second, the *H. mississippiense* proteolytic activity might have also evolved in an African clade through parallel mutation or introgression. A third possibility is that other species of *Histoplasma* (*H. ohiense*, *H. capsulatum ss*, and *H. suramericanum*) lost the serine proteinase activity independently. If that is the case, these two species should harbor serine proteinase pseudogenes. Now that species boundaries have been identified in *Histoplasma*, studies dissecting the processes that lead to serine proteinases in this genus of fungi are within reach.

Yeast colony morphology is arguably the most systematically studied phenotypic difference in *Histoplasma*. The existence of smooth and rough colony morphology in *Histoplasma* was first reported as early as 1987 (50). Genetic analyses suggested that the smooth phenotype was exclusive to a cluster of genotypes (RFLP2, NAm 2), now dubbed *H. ohiense*. Detailed studies of the cell wall with transmission electron microscopy demonstrated that reference strains of *H. ohiense* and *H. capsulatum* differ in their cell wall thickness, with *H. capsulatum* yeast cells showing a greater cell wall thickness compared with *H. ohiense* and that *AGS1* expression is dispensable for *H. ohiense* virulence (26). α-(1, 3)-glucan is required for virulence in *H. capsulatum* and *H. mississippiense* (references 25 and 52, respectively); smooth mutants become avirulent once they are unable to produce this polysaccharide and can be obtained by genetic methods or repeated passages in the lab (25, 50, 51, 60). The *H. mississippiense* Downs isolate showed a smooth colony morphology, probably because it was isolated over 50 years ago and has since been continuously passaged in laboratory conditions. Just like any *Histoplasma* smooth mutant, the Downs isolate has also been shown to be avirulent in mice (49). *H. ohiense* has smooth colonies and lacks α-(1, 3)-glucan, however, it remains virulent. The dissection of the genetic basis of differences in virulence between *Histoplasma* species is a prime example of the importance of understanding species boundaries in eukaryotic pathogens.

There is extensive precedent that once fungal species are identified, phenotypic differences between the newfound taxa are subsequently found. In the case of *Coccidioides—*the first fungal pathogen to undergo a taxonomic revision (61–63)*—*the two different species, *C. posadasii* and *C. immitis,* show differences in thermotolerance, which might be of importance for spherule-to-mycelium transformation and in determining their geographic range (64). Similarly, different species of *Paracoccidioides* show differences not only in antifungal resistance (65) and yeast morphology (66) but also in the host response they elicit in their mammalian hosts (67–70). Although reports of phenotypic variability existed in these fungi (e.g., reference 51), ascribing these differences to species boundaries was only possible once isolated lineages were described in genera that were considered monotypic for almost 100 years.

Our study focuses on five lineages identified through genome sequencing, but there is precedent suggesting that *Histoplasma* contains additional differentiated lineages. Surveys using multilocus-sequence typing reported the existence of over a dozen lineages that might fulfill the criteria for phylogenetic species (27, 71). Sequencing of samples from other locations has revealed additional clades that fulfill the requirements to be considered monophyletic species (Rio de Janeiro in Brazil [29]; India [30]). Genomic studies that quantify the different trajectories along the genome in a worldwide sample couple with phenotypic surveys are sorely needed. Multiple studies have reported inter-isolate differences in the *Histoplasma* genus, but a systematic survey that includes not only reference isolates but also a variety of other strains is needed. For example, the reference isolate of *H. ohiense* (G217B) is more virulent than its counterpart in *H. mississippiense* (WU24) in mouse inoculations (52). Similarly, a clinical isolate of *H. mississippiense* is more resistant to fluconazole than the reference isolate of *H. ohiense* (72, 73), highlighting the importance of considering which species is responsible for causing disease in a patient when deciding on the course of treatment. Finally, the reference strain of *H. capsulatum* (G186A) induces a higher infiltration of monocytic cells in the lungs of mice inoculated with a low dose (10^3^ yeast) than the representative isolates of *H. mississippiense* and *H. ohiense* (52, 74). All these surveys suffer from the same shortcoming, which is that differences between isolates might not be representative of the differences among species. This limitation also applies to our own study as the sample sizes are not sufficient to fully characterize the extent of phenotypic variance in the different *Histoplasma* species. Nonetheless, they are powerful starting points to propel surveys that quantify the extent of inter- and intra-species variation.

The case of *Histoplasma* will require a more systematic exploration than that of *Coccidioides* or *Paracoccidioides* because the number of lineages in *Histoplasma* appears to be much higher than in either of those other fungal pathogens (e.g., additional unsampled lineages or cryptic species could remain unidentified). There is already indication that other unnamed *Histoplasma* lineages show important phenotypic differences. For example, a phylogenetic species restricted to Rio de Janeiro, Brazil, seems to have a higher likelihood of causing hemorrhages than other genotypes (29). Nonetheless, this lineage and the one identified in India are still to be phenotypically characterized. The phenotypic traits we evaluated can certainly aid in the identification of the five species analyzed in this study, but whether they can also be applied to differentiate additional known cryptic species within the *Histoplasma* genus remains to be determined. It is imperative as we define species boundaries that we also make a systematic effort to find phenotypic traits to aid species identification, as they can become useful tools in the clinical setting and could have an impact on the type of antifungal therapy used to treat infections. Our work demonstrates that morphological differences among *Histoplasma* species do exist and provides a blueprint for future surveys.

## Data Availability

Data and analytical code are available in FigShare: https://doi.org/10.6084/m9.figshare.25848820.v1.

## SUPPLEMENTAL MATERIAL

Supplemental Material msphere.00009-24-s0001.docx Figures S1 and S2; Tables S1-S4.

ASM does not own the copyrights to Supplemental Material that may be linked to, or accessed through, an article. The authors have granted ASM a non-exclusive, world-wide license to publish the Supplemental Material files. Please contact the corresponding author directly for reuse.
