# Supplementary material for: Phenotypic characterization of cryptic species in the fungal pathogen Histoplasma
Source: mSphere. 2024 May 21;9(6):e00009-24. doi: 10.1128/msphere.00009-24 (PMC11332167; doi:10.1128/msphere.00009-24)
Supplement: Supplemental Material — Figures S1 and S2; Tables S1-S4. [file msphere.00009-24-s0001.docx]

**SUPPLEMENTARY MATERIAL**

**TABLE S1. *Histoplasma* Isolates used in this study.**

| **Isolate** | **Phylogenetic species *sensu* Kasuga et al. (2003)** | **Phylogenetic species *sensu* Sepúlveda et al. (2017)** | **Origin** | **Year collected** | **OD and cell size** | **Other Designations** |
| --- | --- | --- | --- | --- | --- | --- |
| G186A | H81 lineage | *H. capsulatum* | Panama | 1967 or before | Yes | ATCC 26029  CBS145494 |
| G184A | H81 lineage | *H. capsulatum* | Panama | 1967 or before | Yes | ATCC 26027 |
| G217B | NAm 2 | *H. ohiense* | Louisiana/USA | 1973 or before | Yes | ATCC 26032  CBS145495 |
| CI_17 | NAm 2 | *H. ohiense* | Missouri/USA | Unknown | Yes | CBS145496 |
| WU24 | NAm 1 | *H. mississippiense* | Missouri/USA | 2004 | Yes | CBS145497 |
| CI_19 | NAm 1 | *H. mississippiense* | Missouri/USA | 2003 | Yes | CBS145498 |
| 3_11G | N/A | *H. suramericanum* | Guatemala | 2011 | Yes | CBS145499 |
| 21_14 | N/A | *H. suramericanum* | Guatemala | 2014 | Yes | N/A |
| 27_14 | N/A | *H. suramericanum* | Guatemala | 2014 | Yes | N/A |
| *H. duboisii* | Africa | *H.capsulatum var. duboisii* | ? | Unknown | Yes | N/A |
| H88 | Africa | N/A | Belgium, probably from a former Belgian colony | 1975 or before | Yes | ATCC 32281  RV26821 |
| H143 | Africa | N/A | South Africa | 1954 | yes | CBS 287.54 |
| G222B | NAm 2 | *H. ohiensis* | Louisiana/USA | Unknown | Yes | NA |
| CI_10 | NAm 2 | *H. ohiensis* | Missouri/USA | 2004 |  | NA |
| CI_4 | NAm 2 | *H. ohiensis* | Missouri/USA | 2003 |  | NA |
| CI_9 | NAm 2 | *H. ohiensis* | Missouri/USA | 2004 |  | NA |
| CI_30 | NAm 2 | *H. ohiensis* | Missouri/USA | 1997 |  | NA |
| CI_18 | NAm 2 | *H. ohiensis* | Missouri/USA | 2003 |  | NA |
| CI_6 | NAm 2 | *H. ohiensis* | Missouri/USA | Unknown |  | NA |
| CI_35 | NAm 2 | *H. ohiensis* | Missouri/USA | 2001 | Yes | NA |
| CI_24 | NAm 1 | *H. mississippiensis* | Missouri/USA | Unknown | Yes | NA |
| CI_43 | NAm 1 | *H. mississippiensis* | Missouri/USA | Unknown | Yes | NA |
| CI_22 | NAm 1 | *H. mississippiensis* | Missouri/USA | 2004 |  | NA |
| CI_7 | NAm 1 | *H. mississippiensis* | Missouri/USA | Unknown | Yes | NA |
| CI_42 | NAm 1 | *H. mississippiensis* | Missouri/USA | Unknown |  | NA |
| DOWNS | NAm 1 | *H. mississippiensis* | Missouri/USA | 1968 |  | NA |
| UCLA_531 | NAm 1 | *H. mississippiensis* | California/USA | 1990 or before |  | NA |

**TABLE S2.** AIC values for the best fitting dose-response and linear functions for the growth curve of each *Histoplasma* species.

|  | **AIC** | |
| --- | --- | --- |
| **Species** | **Linear** | **Dose-response** |
| *H. suramericanum* | -5.038041 | -20.2201 |
| *H. ohiense* | 16.69309 | -8.40372 |
| *H. mississippiense* | 9.61138 | -8.56707 |
| *H. capsulatum ss* | 13.66652 | -41.2338 |
| Africa | 1.258701 | -71.5962 |

**TABLE S3. Effect of different optimization values for the dose-response functions.**

| **Species** | **Starting Parameters** | | **AIC** | **Inferred Parameters** |
| --- | --- | --- | --- | --- |
| *H. suramericanum* | *a*=0.97, *b*=10, *c*=100, *d*=1.8 | | -20.22012 | *a* 0.05461 |
|  |  |  |  | *b* 10.00000 |
|  |  |  |  | *c* 99.70641 |
|  |  |  |  | *d* 1.54093 |
| *H. suramericanum* | *a*=0.97, *b*=10, *c*=10, *d*=1.8 | | 38.83496 | *a* 0.4198 |
|  |  |  |  | *b* 10.0000 |
|  |  |  |  | *c* 160.0000 |
|  |  |  |  | *d* 2.0000 |
| *H. suramericanum* | *a*=0.97, *b*=12, *c*=20, *d*=2 | | -20.22012 | *a* 0.05460 |
|  |  |  |  | *b* 10.00000 |
|  |  |  |  | *c* 99.70616 |
|  |  |  |  | *d* 1.54093 |
| *H. ohiense* | *a*=0.97, *b*=10, *c*=30, *d*=2 | | -8.403715 | *a* -0.01128 |
|  |  |  |  | *b* 5.13518 |
|  |  |  |  | *c* 76.21559 |
|  |  |  |  | *d* 2.26046 |
| *H. ohiense* | *a*=0, *b*=50, *c*=80, *d*=1 | | -8.403715 | *a* -0.01128 |
|  |  |  |  | *b* 5.13521 |
|  |  |  |  | *c* 76.21554 |
|  |  |  |  | *d* 2.26045 |
| *H. ohiense* | *a*=0.5, *b*=50, *c*=180, *d*=1 | | -8.403715 | *a* -0.01128 |
|  |  |  |  | *b* 5.13516 |
|  |  |  |  | *c* 76.21556 |
|  |  |  |  | *d* 2.26046 |
| *H. mississippiense* | *a*=.97, *b*=10, *c*=30, *d*=6 | | -8.567065 | *a* 0.00401 |
|  |  |  |  | *b* 5.58656 |
|  |  |  |  | *c* 110.24761 |
|  |  |  |  | *d* 2.08720 |
| *H. mississippiense* | *a*=.07, *b*=10, *c*=130, *d*=6 | | -8.567065 | *a* 0.00401 |
|  |  |  |  | *b* 5.58656 |
|  |  |  |  | *c* 110.24761 |
|  |  |  |  | *d* 2.08720 |
| *H. mississippiense* | *a*=1, *b*=50, *c*=130, *d*=2 | | -8.567065 | *a* 0.00401 |
|  |  |  |  | *b* 5.58656 |
|  |  |  |  | *c* 110.24761 |
|  |  |  |  | *d* 2.08720 |
| *H. capsulatum* ss | *a*=0, *b*=1, *c*=50, *d*=2 | | -41.2338 | *a* 9.231e-05 |
|  |  |  |  | *b* 5.150 |
|  |  |  |  | *c* 7.449e+01 |
|  |  |  |  | *d* 2.138e+00 |
| *H. capsulatum* ss | *a*=0, *b*=10, *c*=100, *d*=2 | | -30.06304 | *a* 0.11000 |
|  |  |  |  | *b* 5.76204 |
|  |  |  |  | *c* 76.18363 |
|  |  |  |  | *d* 2.11889 |
| *H. capsulatum* ss | *a*=0, *b*=10, *c*=150, *d*=2 | | -13.03278 | *a* -0.06824 |
|  |  |  |  | *b* 3.02782 |
|  |  |  |  | *c* 81.79755 |
|  |  |  |  | *d* 2.50000 |
| Africa | *a*=6, *b*=4, *c*=30, *d*=3 | | -33.68027 | *a* 6.738e-03 |
|  |  |  |  | *b* 3.384e+00 |
|  |  |  |  | *c* 1.000e+02 |
|  |  |  |  | *d* 2.207e+00 |
| Africa | *a*=1, *b*=4, *c*=30, *d*=3 | | -71.59624 | *a* 0.002906 |
|  |  |  |  | *b* 5.196654 |
|  |  |  |  | *c* 86.218632 |
|  |  |  |  | *d* 1.870503 |
| Africa | *a*=1, *b*=40, *c*=30, *d*=3 | | -4.478411 | *a* -0.1266 |
|  |  |  |  | *b* 1.5113 |
|  |  |  |  | *c* 200.0000 |
|  |  |  |  | *d* 4.4355 |

**TABLE S4. Contribution of each of the four scored traits to the four principal components.** OD264: Optical density of liquid culture 264 hours post-inoculation.

|  | **DIM1** | **DIM2** | **DIM3** | **DIM4** |
| --- | --- | --- | --- | --- |
| **Halo size** | 8.461 | 54.912 | 35.535 | 1.093 |
| **Cell size** | 12.815 | 45.010 | 40.854 | 1.320 |
| **Colony morphology** | 40.344 | 0.035 | 4.094 | 55.527 |
| **OD264** | 38.379 | 0.043 | 19.517 | 42.060 |

**FIGURE S1. Scree plot showing the percentage of variance explained by each principal component.**

**FIGURE S2. PCR and restriction enzyme diagnostic assay to discriminate among *Histoplasma* spp.** 1.5 Kb PCR fragments of the delta-9 fatty acid desaturase gene from several clinical isolates belonging to *H. ohiense* or *H. mississipiense* were digested with BamHI, XhoI, StuI, BsrGI and BanII. Restriction patterns allow for the identification of the different *Histoplasma* species. Blue: *H. ohiense* (1. G222B, 2. CI#4, 3. CI#6, 4. CI#9, 5. CI#10, 5. CI#18, 7. CI#30, 8. CI#35); Green: *H. missiissiipiense* (9. CI#7, 10. CI#22, 11. CI#24, 12. CI#42, 13. CI#43, 14. Downs, 15. UCLA-531); Magenta: Africa (16. *H. duboisii*); DNA ladder (L. Quick-Load 1 kb Extend DNA Ladder, NEB).
